# Are adenoviruses zoonotic? A systematic review of the evidence

**DOI:** 10.1080/22221751.2019.1690953

**Published:** 2019-11-21

**Authors:** Laura K. Borkenhagen, Jane K. Fieldhouse, Donald Seto, Gregory C. Gray

**Affiliations:** a Division of Infectious Diseases, School of Medicine and Global Health Institute, Duke University, Durham, NC, USA; b Bioinformatics and Computational Biology Program, School of Systems Biology, George Mason University, Manassas, VA, USA; c Global Health Research Center, Duke Kunshan University, Kunshan, People’s Republic of China; d Program in Emerging Infectious Diseases, Duke-NUS Medical School, Singapore, Singapore

**Keywords:** Adenovirus, zoonotic, anthropozoonosis, zooanthroponosis, cross-species

## Abstract

Adenoviruses (AdVs) are major contributors to clinical illnesses. Novel human and animal AdVs continue to be identified and characterized. Comparative analyses using bioinformatic methods and Omics-based technologies allow insights into how these human pathogens have emerged and their potential for host cross-species transmission. Systematic review of literature published across ProQuest, Pubmed, and Web of Science databases for evidence of adenoviral zoonotic potential identified 589 citations. After removing duplicates, 327 citations were screened for relevance; of which, 74 articles received full-text reviews. Among these, 24 were included here, of which 16 demonstrated evidence of zoonotic transmission of AdVs. These documented instances of AdV crossing host species barriers between humans and non-human primate, bat, feline, swine, canine, ovine, and caprine. Eight studies sought to but did not find evidence of zoonosis. The findings demonstrate substantial evidence suggesting AdVs have previously and will continue crossing host species barriers. These have human health consequences both in terms of novel pathogen emergence and epidemic outbreaks, and of appropriate and safe use of non-human adenoviruses for therapeutics. As routine human clinical diagnostics may miss a novel cross-species adenovirus infection in humans, next generation sequencing or panspecies molecular diagnostics may be necessary to detect such incursions.

## Introduction

Human adenoviruses (HAdVs) were among the first human viral pathogens to be isolated and characterized with their near-simultaneous discoveries by Rowe, et al. in 1953 [1] and Hilleman, et al. in 1954 [2]. Subsequently, HAdVs have increasingly been recognized as a major contributor to clinical illnesses including pneumonia, upper and lower respiratory tract diseases, pharyngitis, bronchiolitis, meningitis, rhinorrhea, hemorrhagic cystitis, hepatitis, conjunctivitis, keratoconjuntivitis, and obesity with certain clinical diseases associated with specific adenoviral species and genotypes. With the genomics era, high-resolution sequence data have led to a better and more accurate view beyond the limited epsilon and gamma antigens used originally for serotyping [3,4]. These nonenveloped double-stranded DNA viruses are transmitted via aerosol particles or droplets, the fecal-oral route, the hand-ocular route, and fomites. HAdV infection may also occur upon reactivation from latency following organ transplantation and hematopoietic stem cell transplantation [5].While illnesses associated with HAdV infection are typically asymptomatic or mild, infants, children, the elderly, and immunocompromised patients are at increased risk for severe disease and death [6]. Furthermore, there have been instances of severe and highly contagious disease associated with several genotypes, including HAdV-4, HAdV-7, and HAdV-14, which have caused acute febrile respiratory disease among military trainees [7–9]. HAdV-14 and other genotypes, including HAdV-3, HAdV-5, HAdV-7, HAdV-11, and HAdV-21, have also been associated with acute respiratory illness in healthy civilian populations [10–13]. It should be noted that while one serotype of HAdV-11, genome typed as “HAdV-11a”, is associated with respiratory disease, all other HAdV-11 genome types are associated with renal disease; with whole genome data, the incongruity of “HAdV-11a”, i.e. a renal pathogen associated with respiratory disease, has been resolved, with the strain renamed genotype HAdV-55. Genomic and bioinformatic analyses indicate it is a “Trojan Horse” virus with a recombinant genome that *serotypes* as a renal pathogen, HAdV-11, but *genotypes* as HAdV-14, a respiratory pathogen [14,15] as it contains much of the genome chassis of parental HAdV-14 [16]. HAdV-55 is a potent, circulating respiratory pathogen reported in several large epidemic outbreaks [17,18], illustrating the prominent role of genome recombination in the genesis of emergent adenoviral pathogens. Another example is HAdV-53, a recombinant exhibiting a “non-pathogen” HAdV-22 signature but is associated with highly contagious epidemic keratoconjunctivitis [19]. Later in this report, a zoonotic human respiratory pathogen, HAdV-4, will be discussed in detail within the context of recombination providing a host adaptation to allow optimal viral replication in the new host [20].

Within the genus *Mastadenovirus*, there were 51 serotypes that are now integrated into the 103 human AdV genotypes described in high resolution using genomics [21]. These are parsed into seven species (A-G) that were originally distinguished by biological, clinical, and restriction enzyme digestion properties and have been reconfirmed using Omics data. In addition to humans, AdVs within the genus *Mastadenovirus* infect a wide range of mammalian hosts including bats, bovines, canines, deer, dolphins, equines, murines, non-human primates (NHPs), ovines, swine, sea lions, skunks, squirrels, and tree shrews. Further, there are AdVs in four other genera within the *Adenoviridae* family that infect avian, reptilian, and fish species- essentially covering all vertebrate species studied to date. As with humans, AdV infections in animals can cause diseases that range from asymptomatic to fatal.

In addition to increased attention to HAdVs due to infection-associated morbidity, the biochemical and clinical characteristics of the virus, including its stability and ability to induce innate and adaptive immune responses in mammals, have made AdVs particularly attractive vectors for vaccines and gene therapy. The use of non-human simian AdVs (SAdVs) [22] to circumvent prior exposure associated immunity in humans is of importance. A recent review of HAdV seroprevalence studies reports that by 2018 there were hundreds of vaccine, gene therapy, and cancer trials using HAdV-based vectors [23].

Given that humans and animals are known hosts to AdVs, it is reasonable to consider the possibility of zoonotic or cross-species transmission of AdVs. Hence, we sought to review literature surrounding the zoonotic potential of adenoviruses. For the purposes of this systematic review, “zoonoses” was considered using a One Health definition, as a “two-way street, with humans infecting animals as well as the other way round” [24]. In instances where we sought to specify the directionality of transmission, we used the term “anthropozonoosis” to describe a pathogen moving from animals to humans, and the term “zooanthroponosis” to describe a pathogen moving from humans to animals. Directionality was assigned empirically based on the species of the host identified in the study and the natural host of the virus in question given the available serologic or virologic data.

## Methods

On November 8, 2018, we conducted a search of literature for evidence that AdV can be transmitted between humans and other animals through ProQuest, Pubmed, and Web of Science. This search followed the Preferred Reporting Items for Systematic Reviews and Meta-Analyses (PRISMA) guidelines. To be captured in the search, the terms “adenovirus” (or genus members including “mastadenovirus”, “atadenovirus”, “aviadenovirus”, “ichtadenovirus”, and “siadenovirus”) along with a term implying transmission of virus between humans and other animals in either direction (e.g. “cross-species”, “interspecies”, “zoonoses”, “anthropozoonoses”, etc) must have been included in the text. The complete search string used was, “((adenovirus* OR mastadenovirus OR atadenovirus OR aviadenovirus OR ichtadenovirus OR siadenovirus) AND (cross-species OR interspecies OR zoono* OR anthropono* OR anthropozoono* OR zooanthropono*)).” Citations were exported from the three databases into Endnote, where duplicates were removed. All abstracts and titles were screened, and relevant articles available in English or French were selected for a full review if they mentioned adenovirus and language indicating an investigation of evidence of cross-species virus transmission. At each step, articles were screened separately by J.K.F. and L.K.B. and then reviewed and discussed until the authors were in agreement with respect to interpretation.

## Results

### Search results

**Figure 1.**
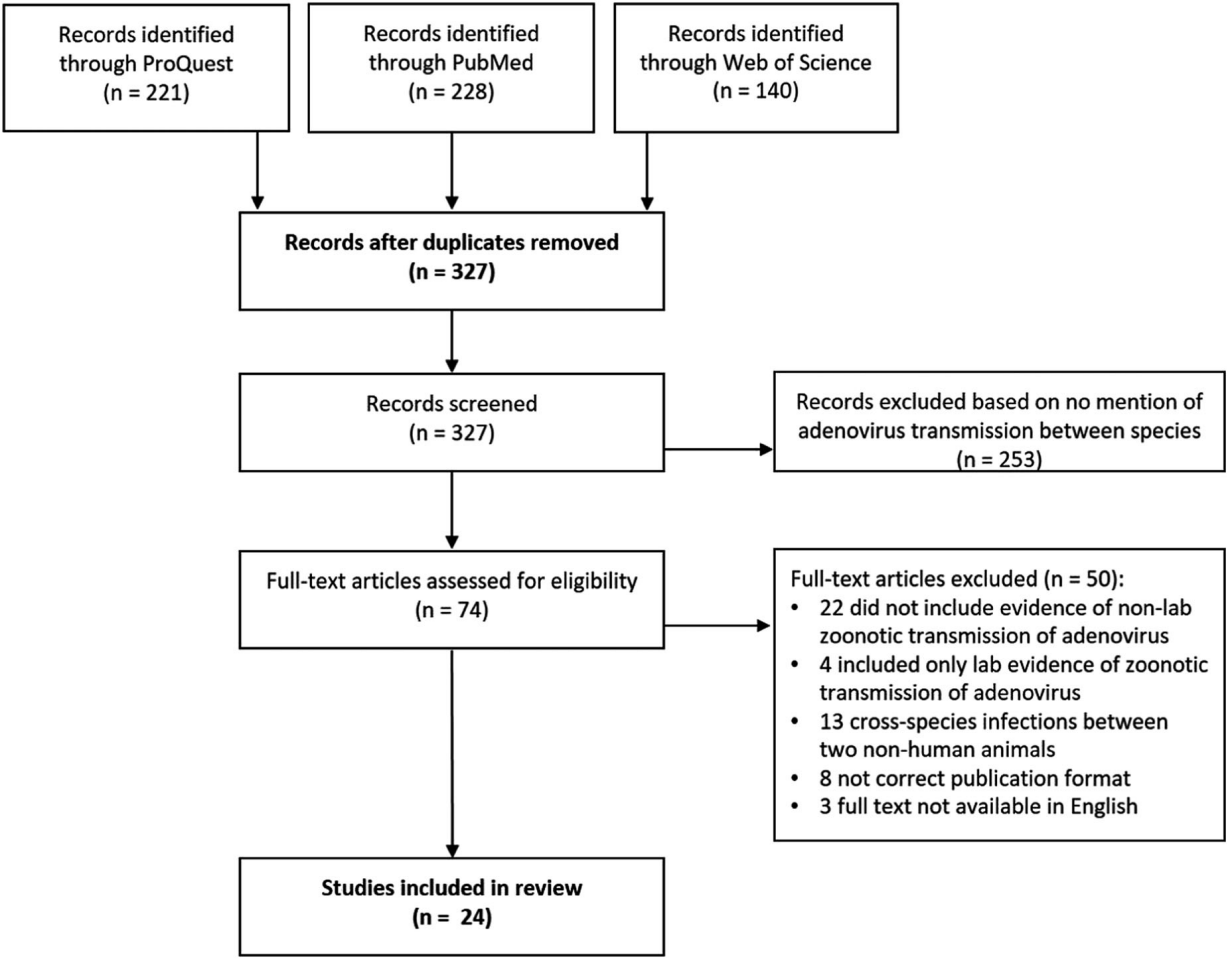
Diagram of article screening process for a systematic review of evidence of adenovirus transmission between humans and other animals. The search captured 327 articles from 3 databases, 24 of which were included in the final review.

The initial search generated 589 citations with 221 through ProQuest, 228 through Pubmed, and 140 through Web of Science (Figure 1). After removing duplicates, the titles and abstracts of the remaining 327 articles were screened for relevance to receive a full review. Following the title and abstract review, 74 articles received a full-text review. Among these, 24 articles were selected for inclusion in the review (Table 1). Sixteen articles (67%) successfully demonstrated evidence of zoonoses and eight articles (33%) sought but failed to demonstrate evidence of zoonoses.

**Table 1.** Publications considered important in evaluating the zoonotic potential of adenoviruses

Evidence of Zoonoses				
Publication		Country and Year	Main Summary	Type of Evidence
1.	Madisch et al. [25]	Global, 2005	Phylogenetic analysis of epitope ε and ץ encoding gene regions of the hexon and fibre genes, respectively, provided evidence of interspecies recombination in both the phylogeny of HAdV-E4 as well as SAdV-E25.	Phylogenetic evidence of zoonosis: ε determinant regions of hexon gene and ץ determinant regions of fibre gene.
2.	Purkayastha et al. [26]	USA, 2005	Genomic analysis of HAdV-4 reveals sequence similarity closer phylogenetic relationship to SAdVs than HAdVs, suggesting evolutionary origins of HAdV-4 as a product of zoonotic transmission; literature supports chimpanzee to human transmission. Recombination or lateral gene transfer of hexon from HAdV-4, presumably of chimpanzee origins, to HAdV-16.	Phylogenetic evidence of anthropozoonosis: complete and annotated genome sequence of HAdV-4.
3.	Phan et al. [27]	Japan, 2006	AdV type 1 with 100% identity and 97% identity to human AdV was detected in 1-year old female with acute gastroenteritis. The HAdV and feline AdV detected belong to the same AdV-1 cluster, prototype Adenoid 71.	Phylogenetic evidence of anthropozoonosis: 100% amino acid sequence homology in seven hypervariable regions of the hexon gene; 97% amino acid sequence homology in fibre genes (BLAST and Clustal X).
4.	Xiang et al. [28]	Cameroon, Côte D`Ivoire, Nigeria, Thailand, USA 2006	Humans in the U.S. had no neutralizing antibodies to chimpanzee AdVs, but human serum from Cameroon and Nigeria showed higher prevalence of antibodies to AdV-C6 and AdV-C1. Samples from Côte D`Ivoire high prevalence of antibodies to AdV-C68 and AdV-C6.	Serological evidence of anthropozoonosis: Sera from humans and chimpanzees were tested for neutralizing antibodies to chimpanzee-derived AdV- C68, -C6 and -C1.
5.	Roy et al. [29]	Global, 2009	Genomic sequencing of different primate species stool samples collected across Africa and the U.S. found a high prevalence of AdV which could be classified into species corresponding with HAdV B, C, and E groups	Phylogenetic evidence of zooanthroponosis: Samples first cultured and analyzed by PCR; 8 genes (hexon, penton base, protease, E1A, DBP, polymerase, pTP).
6.	Wevers et al. [30]	Global, 2011	HAdVs species A-G were detected in fecal, blood and tissue samples of wild or captive NHPs. The majority of human AdVs detected were in HAdV-E (46%), -B (35%), and –C (15%) clades.	Phylogenetic evidence of zooanthroponosis: Pan-primate AdV-specific PCR targeting highly conserved polymerase gene. Near complete sequencing of hexon gene.
7.	Chen et al. [31]	USA, 2011	Researcher at a primate research centre was in contact with monkey infected with TMAdV and developed acute respiratory illness. Two family members also developed ILI 1–2 weeks after researcher fell ill. Samples from the researcher and one ill family member were found positive for neutralizing Abs to TMAdV.	Serological evidence of anthropozoonosis: Four months after outbreak for researcher with direct contact and one year after outbreak for family members: Ab titre for researcher was 1:32 and for family member 1:8. Phylogenetic: Whole-genome sequencing found the TMAdV distinct from known HAdVs. Closest simian AdV relative is SAdV-3, -18 and -21.
8.	Roy et al. [32]	USA, 2012	Fecal sample from rhesus macaques from 5 primate facilities found 23 SAdV isolates. Sequencing revealed SAdV-18 related to HAdV F.	Phylogenetic evidence of zooanthroponosis: whole genome sequencing.
9.	Baker et al. [33]	Ghana, 2013	An *Eidolon helvum* (fruit bat) AdV-1 collected from urine yields a hexon protein sequence that clades near the HAdV clade, and shares 77% and 90% amino acid similarity across 58 and 63 amino acids, respectively, spanning the 900 amino acid hexon proteins.	Phylogenetic evidence of zooanthroponosis: Hexon sequence similarity across 58 and 63 amino acids, respectively, of the 900 amino acid protein.
10.	Chiu et al. [34]	USA, 2013	Following outbreak of four baboon AdVs at primate research facility, six facility personnel were found to have neutralizing Abs to BaADV-2 and -4 and five also had evidence of neutralizing Abs titres to BaAdV-1. Staff anecdotally reported ILI. None found to have neutralizing Abs to BaAdV-3. Five children under 5 years of age also tested as controls, all of whom were negative for neutralizing Abs to BaAdV -1, -2, -3, and -4.	Serological evidence of anthropozoonosis: Researcher with most contact with ill baboons had the highest neutralizing Ab response of 1:128.
11.	Dehghan el al. [35]	USA, 2013	From a comparative genomic analysis, SAdV type 35 (isolated by Roy et al. 2009) yielded similar identities with HAdV-B21 (89.7%); HAdV-B50 (89.2%) HAdV-B16 (87.8%); and HAdV-B7 (85.5%). A sequence recombination analysis also demonstrated an example of recombination between HAdVs and SAdVs.	Phylogenetic evidence of zoonosis: Whole genome and select gene sequences using BLAST, MAFFT and MEGA4 software; amino acid sequence analysis and nucleotide sequence analysis
12.	Dehghan el al. [20]	USA, 2013	Recombination or lateral DNA sequence transfer of ITR, and specifically the NF-1 viral replication protein binding site, from a HAdV-B species virus to HAdV-4, as host adaptation for optimal viral replication in human cells.	Phylogenetic evidence of anthropozoonosis: Whole genome sequencing; Sequence alignment and phylogenetics for the ITR and viral replication protein binding sequence.
13.	Hoppe et al. [36]	DRC, Rwanda, Uganda, 2015	Detected HAdV-B in seven wild gorilla samples; Sample from Eastern lowland gorilla in DRC found near complete genome sequence of HAdV-B. Evidence of intra-species recombination between HAdV-B obtained from wild gorillas and chimpanzees.	Phylogenetic evidence of zooanthroponosis: nearly full genome sequences, amplified widely overlapping PCR fragments with 19 sets of degenerate nested primers and long-distance PCR.
14.	Hoppe et al. [37]	Cameroon, CAR, Côte D`Ivoire, DRC, Gabon, Rwanda, Uganda 2015	HAdV-B -C -E and -F were detected by PCR in fecal samples from wild great apes (gorillas, bonobos and chimpanzees).	Phylogenetic evidence of zooanthroponosis: amplified block with V, pX, pVI and hexon genes, polymerase sequences, V gene using BLAST.
15.	Pauly et al. [38]	Côte D`Ivoire, 2015	A study of humans and domestic animals found no animal AdV in human feces but several HAdV types were detected in animal fecal samples and rectum swabs from pigs, dogs, goats and sheep.	Phylogenetic evidence of zooanthroponosis: PCR targeting hexon gene and phylogenetic analysis using BLAST.
16.	Sukmak et al. [39]	Thailand, 2017	Fecal samples of macaques found evidence of three AdVs, with two clustering in HAdV-G cluster, demonstrating close relationship to HAdV-52, supporting hypothesis HAdV-52 was originally a SAdV.	Phylogenetic evidence of zooanthroponosis: hexon and polymerase genes.
17.	*Dehghan et al. [40]	USA, 2019	From a comparative genomic analysis, HAdV-76 is nearly identical to SAdV type 35.1 and 35.2 (isolated by Roy et al. 2009), with whole genome sequence noted as 99.5% and 99.6% identical to genomes from SAdV-B35.1 and SAdV-B35.2, respectively. A sequence recombination analysis also demonstrated an example of recombination between HAdVs and SAdVs.	Phylogenetic evidence of zoonosis: whole genome sequencing; sequence alignment and phylogenetic analysis.
**Lack of Evidence of Zoonoses**				
**Publication**		**Country and Year**	**Main Summary**	**Type of Evidence**
18.	Miller et al. [41]	USA, 1971	A serological study of a group of poultry-exposed persons and a control group of unexposed persons found no seropositivity in either group for avian AdV	Serological: neutralization in ovo (CELO virus), and neutralization by plaque reduction.
19.	Kayali et al. [42]	USA, 2009	The study found no serological evidence that exposed turkey workers were infected with hemorrhagic enteritis viruses (avian adenoviruses)	Serological: HEV antibody test kit used to test sera of exposed turkey workers and controls with no exposure; HAdV-2, -3, -41 also tested to control for cross-reactivity
20.	Li et al. [43]	USA, 2010	An analysis of bat guano for AdV among other viruses found “no close homologue of a known human viral pathogen” through phylogenetic evidence.	Phylogenetic: metagenomic analysis using second generation sequencing.
21.	Phan et al. [44]	USA, 2011	A characterization of fecal samples from rodents detected sequences of a novel AdV in a deer mouse, which clustered with murine AdV-2 but no genetic similarities with HAdVs.	Phylogenetic: two separate contigs encoding 10% of AdV Hexon protein
22.	Nkogue et al. [45]	Gabon, 2016	Surveillance of AdV infection in fecal samples from western lowland gorillas and humans residing in close proximity revealed a high prevalence of AdV in gorillas and humans. HAdV-B, -C, -and E were detected in gorillas. HAdV-C, and -D were detected in humans; however, the HAdV-C genes in humans and gorillas wer genetically distinct.	Phylogenetic: nested PCR targeting DPOL and hexon genes. Nucleotide sequences of amplicons confirmed using BLAST
23.	Zheng et al. [46]	China, 2016	Rectal swabs from bats and stool samples from outpatients with diarrhea were analyzed for bat and human AdVs. All bat and human AdVs detected were mastadenoviruses but only 57.1–69.3% aa similarity was detected between the two.	Phylogenetic: nested PCR targeting DPOL gene. Sequencing using BioEdit and comparison using BLAST
24.	Wang et al. [47]	USA, 2018	Nasal swabs from human subjects with influenza like illness who were living in close proximity to dense poultry and swine farming operations were examined for evidence of panspecies adenoviruses. No evidence of zoonotic adenoviruses was found.	Molecular: PCR targeting hexon gene
25.	Dadáková et al. [48]	Tanzania, 2018	Fecal samples from unhabituated eastern chimpanzees demonstrated HAdV-B, -C and -E strains; however, all were distinct from human, even gorilla and bonobo clusters, indicating strict host specificity of the viruses	Phylogenetic: PCR targeting DPOL gene partial DPOL and/or hexon sequencing; ClustalW amino acid alignment; BLAST analysis and hexon-based phylogenetic analysis

aa = amino acid; Abs = antibodies; AdV = adenovirus; AdV-2 = adenovirus serotype 2; AdV-C1 = adenovirus species C serotype 1; AdV-C6 = adenovirus species C serotype 6; AdV-C68 = adenovirus species C serotype 68; BaAdV = baboon adenovirus; BaAdV -1, -2, -3, and -4 = baboon adenoviruses serotypes 1, 2, 3 and 4; BLAST = Basic Local Alignment Search Tool; CAR = Central African Republic; CELO = chicken embryo lethal orphan; DBP = DNA-binding protein; DPOL = DNA polymerase gene; DRC = Democratic Republic of the Congo; HAdV = human adenovirus; HAdV-2 = human adenovirus serotype 2; HAdV-3 = human adenovirus serotype 3; HAdV-4 = human adenovirus serotype 4; HAdV-41 = human adenovirus serotype 41; HAdV-52 = human adenovirus serotype 52; HAdV-B = human adenovirus species B; HAdV-B7 = human adenovirus species B serotype 7; HAdV-B16 = human adenovirus species B serotype 16; HAdV-B21 = human adenovirus species B serotype 21; HAdV-B50 human adenovirus species B serotype 50; HAdV-C = human adenovirus species C; HAdV -E = human adenovirus species E; HAdV-E4 = human adenovirus species E serotype 4; HAdV-F = human adenovirus species F; HAdV-G = human adenovirus species G; ILI = influenza-like-illness; ITR = inverted terminal repeat; MAFFT = multiple alignment using fast Fourier transform; MEGA4 = Molecular Evolutionary Genetics Analysis version 4.0; PCR = polymerase chain reaction; pTP = terminal protein precursor; SAdV = simian adenovirus; SAdV-E25 = simian adenovirus species E serotype 25; TMAdV = titi monkey adenovirus.

*Note that Dehghan et al. [40] was published after the systematic review search, but has been included in this table due to relevance to the review.

### Strength of evidence

Of the 24 studies, 18 (75%) provided phylogenetic evidence, four (17%) provided serological evidence, one provided both phylogenetic and serological data, and one relied on molecular evidence supporting or refuting the zoonotic potential of the viruses. Of the studies presenting phylogenetic evidence of zoonoses, three demonstrated interspecies recombination of HAdVs and simian AdVs (SAdVs) [20,25,35] and one found evidence of an intraspecies recombination between HAdV-B detected in wild gorillas and chimpanzees [36]. Ten (10) studies used genomic sequencing to identify human or non-human AdV species in biological samples.

The study by Chen, et al. provided both phylogenetic evidence using whole-genome sequencing and serological evidence of human infections with a titi monkey adenovirus (TMAdV) producing arguably the strongest evidence of a anthropozoonotic transmission of adenoviruses [31]. In this study, a researcher at a California primate centre who had been in closest contact with a monkey infected with TMAdV developed an acute respiratory illness and had a convalescent serum sample that was seropositive for TMAdV [antibody (Ab) titre 1:32]. Subsequently, two members of this researcher’s family developed influenza-like-illnesses one to two weeks after the researcher initially fell ill; and a year later, one of those family members was also found positive for neutralizing antibodies to TMAdV (Ab titre 1:8). The TMAdV was a novel adenovirus, distinct from all species A-G HAdVs and with only 54% to 56.3% identity with the closest SAdV relatives, SAdV-3, -18, and -21.

Another report supporting anthropozoonotic transmission of adenoviruses to humans from primates noted a 1997 outbreak of acute respiratory illness in baboons at a primate research facility in Texas. In this outbreak, six novel baboons adenoviruses (BaAdVs) comprising a proposed novel “simian adenovirus C (SAdV-C)” species were isolated from asymptomatic and sick baboons, including two fatalities. Although no humans showed symptoms, antibodies to these new BaAdVs were found in staff personnel at the facility, as well as in other baboons [34].

DNA sequencing data show genomic “footprints” of cross-species transmission as two nearly identical SAdVs, isolated independently from a chimpanzee at the New Iberia Research Center (SAdV-B35.1; University of Louisiana; Lafayette, LA) and a bonobo in a zoological setting (SAdV-B35.2; San Diego Zoo; San Diego, CA) by Roy, et al. [29], contain recombinant genomes with contributions from SAdV-21 (chimpanzee), HAdV-21(human), SAdV-27.1 (chimpanzee), and HAdV-16 (human) [35]. Interestingly, an emergent human pathogen, HAdV-76, with a nearly identical genome [40] to both SAdV-35.1 and -35.2 was associated with a respiratory disease fatality in Texas in 1967 [49].

### Evidence of anthropozoonoses

We identified six studies (25%) demonstrating evidence of anthropozoonoses, five (21%) of which present transmission of AdVs from NHPs to humans. In one of these studies, Xiang et al. presented serological evidence of anthropozoonotic transmission of AdV to NHPs from five countries across three continents [28]. The study found humans in Côte D`Ivoire, Cameroon, and Nigera with neutralizing antibodies for several chimpanzee-derived AdV-C; however, none of the 50 humans working in U.S. zoos in close contact with primates had detectable antibodies.

Significantly, one of the first human viral pathogens isolated and characterized in 1953 as cytopathogenic respiratory illness agent RI-67 [2], later renamed as HAdV-4, has been shown to contain a genome with near identity to several chimpanzee adenoviruses [20,26], which suggests anthropozoonosis. Prior to the Omics era, it was speculated that this unique and odd human adenovirus, HAd4, was more closely related to the few chimpanzee adenoviruses characterized and was grouped in its own taxonomic clade, as species E [50]. It was thought to be the “archetype”, having limited amino acid and nucleic acid sequence similarities to human adenoviruses of species B and C [50,51]. Additional and more whole genome DNA sequences from the dataset including all human adenovirus genotypes and more simian adenoviruses [20,26] suggest a chimpanzee origin for this still-significant human pathogen [52]. A long-standing curiosity of HAdV-4 was its apparent host range limitation to the U.S. military basic trainee population [52]. A study by Dehghan et al. found that two recent HAdV-4 isolates from U.S. military trainees shared genome near identities with five simian AdV genomes isolated from chimpanzees [20]. Furthermore, unlike the AdV-4 prototype and vaccine field strains [26,53], these recent circulating isolates contain a modified Inverted Terminal Repeat (ITR) sequence [20], apparently recombined from a parental human adenovirus, that provides all three viral replication factor binding sites that have been reported as required for optimal replication in human cells [54–58]. HAdV-4 outbreaks have been recently reported globally and in civilian populations [59,60]; DNA sequencing reveals the recombinant ITR [59].

Another study by Phan et al. reported RT–PCR detection of feline AdV in a one-year old child hospitalized for acute gastroenteritis [27]. Through sequencing, the specimen was identified as HAdV with 100% amino acid sequence identity in seven hypervariable regions of the hexon gene and 97% in fibre genes (BLAST and CLUSTAL X).

### Evidence of zooanthroponoses

The review found eight studies (33%) demonstrating evidence of zooanthroponoses, including six studies (25%) that provided evidence of transmission of AdVs from humans to NHPs. Across those six studies, the most common HAdV species detected in NHPs were HAdV-B, -C, -E, and -F, though the global study of fecal, blood and tissue samples from wild and captive NHPs conducted by Wevers et al. found all HAdVs species A-G [30].

Baker et al. isolated a novel AdV from bat urine in Ghana and sequenced the hexon gene [33]. This *Eidolon helvum* or fruit bat AdV-1 hexon forms a phylogenetic clade separate from previously identified bat AdVs, and is distinct in that it clusters with the HAdV clade. The high bootstrap value of 93 in their phylogenetic analysis indicates strong support that will likely be preserved with additional sampling. It was noted by Baker et al. that the hexon shared 77% and 90% amino acid similarities with the HAdV counterpart across 58 and 63 amino acids, respectively, of the 900 amino acid hexon protein [33].

Additionally, Pauly et al. detected HAdV in fecal samples collected from pigs, dogs, sheep, and goats in Côte d’Ivoire [38]. Even more intriguing is the isolation of a HAdV-1-like virus from cats, following a large sample screening of domestic cats using a HAdV-1 hexon antigen and ELISA in the mid-1990s by Ongradi et al. [61]. Sequence determination of the feline hexon and fibre genes showed near-identity to the human counterparts [62]. This virus was reported to replicate in both human and monkey cells in vitro [61], which was updated recently (2019) to include a screen of various permissive and non-permissive human and other animal cells, along with additional immunochemical analyses [62].

### Studies Lacking evidence of zoonoses

The review captured eight studies which did not find evidence of the zoonotic potential of AdVs. Of those studies, four specifically sought to find evidence of zoonanthroponoses and three specifically sought to find evidence of anthropozoonoses. Five studies provided phylogenetic evidence characterizing fecal samples from bats, rodents or NHPs, determining that the AdVs detected were genetically distinct from HAdVs. One negative study, Wang et al., was premised on the absence of molecular presence of AdV [47]. The Nkogue et al. study of western lowland gorillas and humans in Gabon concluded detected HAdV-C in both hosts. However, characterization of the HAdV-C species detected in the human and gorilla samples were genetically distinct, leading Nkogue and colleagues to interpret this as a lack of evidence for zoonoses [45].

## Discussion

Unlike our previously published systematic review of the zoonotic potential of enteroviruses (EVs) [63], in which nearly half of the publications included in the final review were clustered in Central Africa, the studies included in this review varied geographically. Of the 16 publications demonstrating phylogentic and/or serological evidence of zoonotic adenovirus transmission, four studies were conducted globally with samples spanning three or more continents, four were conducted across several countries in West and Central Africa, six studies were conducted in the United States, one study was conducted in Japan, and one in Thailand.

Of the negative studies, five were conducted in the United States, and one each in China, Gabon, and Tanzania. While this regional distribution may suggest the zoonotic potential of adenoviruses is not geographically isolated or clustered, it is important to note that the two U.S.-based studies demonstrating zooanthroponosis were based in primate facilities [31,34]. Additionally, the serological study by Xiang et al. examining sera from humans and chimpanzees in Cameroon, Côte D`Ivoire, Nigeria, Thailand, and the United States found neutralizing antibodies in human serum to chimpanzee-derived antibodies were more prevalent in humans living in sub-Saharan Africa than humans living in Thailand and the United States [28].

The majority of the studies identified as providing evidence for zoonotic transmission of AdVs reported species crossing between humans and non-human primates. In addition to these 13 reports, a recent study that was published after the search date for this review by Dehghan et al. unearthed evidence of a unique “ping-pong” pattern of AdV transmission between humans, chimpanzees, and bonobos when conducting genomics analyses of archived AdV sequences [40]. Of note, an emergent recombinant adenovirus typed by the authors, HAdV-B76, was associated with a human fatality in 1965.

Additional relevant studies were published following the initial review in November 2018, including the previously cited Dehghan et al. study [40], Ongrádi et al. study of feline AdV [62], the Zhang et al. study of HAdV-E4 circulating in Hong Kong [59].

This systematic review had several limitations. While a number of studies with minimal or no evidence of zoonotic adenovirus transmission were identified, these negative studies may have been more likely to be missed in this search. Additionally, our search strategy excluded studies with evidence of cross-species infections among nonhuman animals. Indeed, of the 50 articles excluded following the full-text review, 13 were excluded from the final analysis as they strictly investigated cross-species infections between two non-human animals.

## Conclusions

Given the evidence in this review, it is apparent that AdVs have crossed, and will continue to cross host species. This has been especially true for the species barrier between humans and NHPs (and likely between different NHP species), but it may also occur between humans and other animal species. Humans with intense exposure to NHPs, such as though occupational exposure at primate facilities, may be at higher risk of becoming infected with a zoonotic adenovirus. Routine clinical diagnostics are not likely to detect a novel animal adenovirus infection in humans. Hence, clinicians must have a strong degree of suspicion and apply next-generation sequencing and/or panspecies diagnostics (not yet commercially available) in detecting such a novel strain. Wellehan et al [64] have reported a conventional panspecies PCR approach, which coupled with limited sequencing, may be so employed.
